# Insights Into MicroRNA-Mediated Regulation of Flowering Time in Cotton Through Small RNA Sequencing

**DOI:** 10.3389/fpls.2022.761244

**Published:** 2022-03-31

**Authors:** Yu Zhou, Aye Aye Myat, Chengzhen Liang, Zhigang Meng, Sandui Guo, Yunxiao Wei, Guoqing Sun, Yuan Wang, Rui Zhang

**Affiliations:** Biotechnology Research Institute, Chinese Academy of Agricultural Sciences, Beijing, China

**Keywords:** cotton, early flowering, transcriptome, miRNA, shoot apex

## Abstract

The timing of flowering is a key determinant for plant reproductive. It has been demonstrated that microRNAs (miRNAs) play an important role in transition from the vegetative to reproductive stage in cotton; however, knowledge remains limited about the regulatory role of miRNAs involved in flowering time regulation in cotton. To elucidate the molecular basis of miRNAs in response to flowering time in cotton, we performed high-throughput small RNA sequencing at the fifth true leaf stage. We identified 56 and 43 miRNAs that were significantly up- and downregulated in two elite early flowering cultivars (EFC) compared with two late flowering cultivars (LFC), respectively. The miRNA targets by RNA sequencing analysis showed that *GhSPL4* in SBP transcription factor family targeted by *GhmiR156* was significantly upregulated in EFCs. Co-expression regulatory network analysis (WGCNA) revealed that *GhSOC1*, *GhAP1*, *GhFD*, *GhCOL3*, and *GhAGL16* act as node genes in the auxin- and gibberellin-mediated flowering time regulatory networks in cotton. Therefore, elucidation of miRNA-mediated flowering time regulatory network will contribute to our understanding of molecular mechanisms underlying flowering time in cotton.

## Introduction

MicroRNAs (miRNAs) are a class of endogenous small non-coding RNA molecules. In plants, miRNAs post-transcriptionally regulate gene expression by mediating the degradation of target mRNAs or by inhibiting the translation of target mRNAs (Fei et al., 2013; Iwakawa and Tomari, 2013; Ren et al., 2014; Song et al., 2019). For example, most miRNAs guide Argonaute (AGO) proteins cleave the target mRNAs on the basis of near-perfect sequence complementarity by forming an RNA-induced silencing complex (RISC; Eamens et al., 2009; Yu et al., 2017). It is now clear that miRNAs play an important role in plant development such as regulation of abiotic stress responses, meristem organization, and leaf morphology and size (Song et al., 2019).

Flowering time is a key trait that is strongly associated with crop yield (Itoh et al., 2010). Strict regulation of flowering time is critical for reproductive success, enabling the completion of plant generation under appropriate environmental conditions (Purugganan and Fuller, 2009). There are several miRNAs that have been shown to function in controlling flowering. For example, miR156 and miR172 regulate the vegetative to reproductive phase transition as part of the age-dependent flowering pathway (Wu et al., 2009). A decline in the miR156 expression level coincides with the upregulation of *SQUAMOSA-PROMOTER BINDING PROTEIN-LIKE* (*SPL*), which activates *LEAFY* (*LFY*), *FRUITFULL* (*FUL*), and *APETALA1* (*AP1*; Yamaguchi et al., 2009). The miR172 represses the expression *APETALA2* (*AP2*) by activation of SPLs, which further represses the floral inducer FT (Mathieu et al., 2009; Xie et al., 2020). In addition to miR156 and miR172, miR159, miR319, and miR399 also play important role in controlling flowering time. The miR159 represses the expression of miR156 by targeting MYB33 which binds to the *LFY* promoter (Gocal et al., 2001), and miR319 regulates the *TEOSINTE BRANCHED/CYCLOIDEA/PCF* (*TCP*) to promote flowering by binding to the *CO* promoter and activating transcription (Liu et al., 2017). MiRNA also plays an important role in flowering regulation of other crops. Monocot-specific miR528 promotes flowering under long-day conditions by targeting RED ANDFAR-RED INSENSITIVE 2 (OsRFI2) in rice (Yang et al., 2019). APETALA2-like gene targeted by miR172 promotes vegetative phase change in maize (Lauter et al., 2005). In *Brachypodium distachyon*, *pooideae*-specific miR5200 targets FT in flowering time regulation (Wu et al., 2013).

Gibberellic signaling pathway widely determines the flowering time in plants (Yamaguchi, 2008). Earlier studies showed that DELLA proteins (DELLAs) are negative regulator of gibberellin target genes such as REPRESSOR OF ga1-3 (RGA), GA-INSENSITIVE (GAI), RGA-LIKE 1 (RGL1), RGL2, and RGL3 (Achard et al., 2009). A recent study showed that DELLAs repress flowering in Arabidopsis through negative regulation in expression of CONSTANS (CO) which induce the expression of FLOWERING LOCUS T (FT; Wang et al., 2016; Xu et al., 2016). Furthermore, the interaction of CO with NF-YB2 is inhibited by the DELLA protein, thus suppressing flowering (Xu et al., 2016). Besides that, gibberellin signaling pathway regulates the DELLAs by crosstalk with auxin and cytokinin signaling pathway. For example, AUX/IAA proteins also repress the stability of DELLAs (Fu and Harberd, 2003). In addition, ARFs and IAAs directly regulate the GA metabolic enzymes such as GA20ox, GA3ox, and GA2ox (O'Neill and Ross, 2002; Frigerio et al., 2006).

Upland cotton (*Gossypium hirsutum* L.) is the most economically valuable textile crop in the world (Paterson et al., 2012). A total of 1,500 miRNAs have been identified in different cotton species,^1^ including 315 miRNAs in *G. arboreum*, 434 in *G. barbadense*, 465 in *G. hirsutum*, and 286 in *G. raimondii*. RNA sequencing (RNA-seq) is a powerful tool to study gene expression and gene regulatory relationships. Recent studies have shown that regions located to chromosome A05 and chromosome D03 are enriched in early maturity traits, and *GhCAL* regulates flowering time by controlling the transition from vegetative to reproductive growth (Ma et al., 2018; Cheng et al., 2020; Li et al., 2021). *GhUCE* and *GhBRC1* regulate cotton flowering by integrating multiple hormone pathways (Ma et al., 2018; Sun et al., 2021). Recent study also demonstrated that auxin signaling-associated miR167 also directly affected the differentiation of floral in cotton (Arora and Chaudhary, 2021).

Although several studies on molecular mechanism on flowering time control have been carried out separately focusing on miRNAs and transcriptome sequencing, association analysis combining both omics methods has not been reported yet. Here, we performed an association analysis for mRNAs and miRNAs in four different varieties to gain a better understanding of gene expression and their regulation of this process.

## Materials and Methods

### Plant Growth and Treatment Conditions

The early-maturing upland cotton cultivars CZ-3 and 4-5-26 and the late-maturing cultivars S25 and 48xi were planted at the Biotechnology Research Institute of the Chinese Academy of Agricultural Sciences Experimental Field in Beijing City, and the plants were managed using general field management practices. The two early-flowering varieties CZ-3 and 4-5-26 belong to different background. However, S25 and 48xi belong to same background. Ten shoot apexes at the fifth true leaf stage were collected for one biological replicate, and two biological replicates were used for RNA extraction. These samples were immediately frozen in liquid nitrogen and stored at −80°C prior to use in the experiments.

### Small RNA Sequencing and Data Analysis

miRNAs were isolated and purified using the RNAprep Pure kit (Tiangen, China) following the manufacturer’s procedure. Raw sequencing reads were processed using an in-house program, ACGT101-miR (LC Sciences, Houston, TX, United States) to remove adapter dimers, bad reads, low complexity reads, common RNA families (rRNA, tRNA, snRNA, and snoRNA), and repeats. After this filtering, the obtained over 7,800,000 valid reads per library were used for further analyses (Supplementary Figure S1). Subsequently, unique sequences with lengths of 18–25 nucleotides were mapped to specific species precursors in miRBase 22.0 by BLAST searches to identify known miRNAs and novel 3p- and 5p-derived miRNAs. The differentially expressed miRNAs were selected using |log2 (fold change)| > 1 and value of *p* < 0.05 thresholds. Target gene identification was performed with Psrobot.^2^ These sequence data have been submitted to the NCBI databases under accession number PRJNA785082.

### Transcriptome Sequencing and Data Analysis

RNA was extracted from two early flowering varieties, CZ-3 and 4-5-26, and two later flowering varieties, S25 and 48xi. Total RNA was isolated and purified using TRIzol reagent (Invitrogen, Carlsbad, CA, United States) following the manufacturer’s instructions. The RNA concentration and purity were determined using a NanoDrop ND-1000 spectrophotometer (NanoDrop, Wilmington, DE, United States). The RNA was sequenced (150-base paired-end reads) using an Illumina Novaseq™ 6000 instrument following the manufacturer’s recommended protocol. After removing the adaptor sequences, duplicated sequences, ambiguous reads, and low-quality reads, a total of 41,509,692 (CZ-3), 44,302,944 (4-5-26), 49,553,584 (S25), and 51,455,996 (48xi) valid reads were used in the analyses (Supplementary Figure S1). Gene expression levels were determined using the fragments per kilobase of transcript per million mapped reads (FPKM) method. The differentially expressed mRNAs were selected with fold change >2 or fold-change <0.5 and value of *p* < 0.05 using the R package edgeR.^3^ Functional annotation of the DEGs was conducted through the Cotton Functional Genomics website^4^ with a significance level of 0.05 and a minimum gene number for each analyzed term = 3. These sequence data have been submitted to the NCBI databases under accession number PRJNA785082.

### Weighted Correlation Network Analysis and Functional Enrichment Analysis

The WGCNA package was used to analyze the co-expressed genes^5^ with all parameters set as defined: “soft_power = 26, minModuleSize = 30 and mergeCutHeight = 0.25.”

### cDNA Preparation and Gene Expression Analysis

miRNA was reverse-transcribed using SuperScript™ II Reverse Transcriptase (Invitrogen, Carlsbad, United States) according to the manufacturer’s instructions. For quantitative real-time PCR (qRT-PCR), SYBR Green I was added to reaction mix and amplifications were performed on a CFX96 Real-Time PCR Detection System (Bio-Rad, Hercules, CA, United States). The ubiquitin gene *GhUBQ7* (Ghir_A11G011460) was used as internal reference control, and the relative expression levels were calculated using the modified 2^−ΔΔCT^ method. Values are means ± SD of three biological replicates. Student’s *t*-test was used for statistical analysis. Asterisks indicate statistically significant differences compared with the wild type (^*^p ≤ 0.05; ^**^p ≤ 0.01).

### Plasmid Construction and Transformation

For cotton transformation, the pCLCrV:VIGS constructs were constructed by a method modified from Gu et al. (2014). The basic constructs, pCLCrVA, pCLCrVB, and CLCrV-CHLI, were obtained from them. The fragment of pre-miR156 and a small tandem target mimic (STTM) sequence containing two imperfect binding sites separated by a 48 bp spacer, both were synthesized (Sangon) and inserted into the plant expression vector pCLCrVA by SpeI and PacI to get OE-miR156 and STTM-miRNA vectors. The above pCLCrVA vectors and pCLCrVB construct were introduced into the Agrobacterium tumefaciens strain EHA105. The Agrobacterium cultures were pelleted and resuspended. After 3 h incubation at room temperature, Agrobacterium strains harboring different pCLCrVAs were mixed with an equal volume of Agrobacterium harboring pCLCrVB separately. The mixed Agrobacterium solutions were infiltrated into the abaxial side of cotyledons of 2-week-aged cotton seedlings by needleless syringes through small wounds. Keep the plants at 24°C for 3 weeks till CLCrV-CHLI plants grew out white leaves; then move all plants to the environment at 28°C under long-day conditions (16 h light/8 h dark).

For Arabidoposis transformation, the fragment of pre-miR399e was synthesized (Sangon) and inserted into the plant expression vector pCAMBIA 2,300 using KpnI and PstI. The floral-dip method was used for transformation (Bent, 2006).

## Results

### Cotton Cultivars Have Distinct Flowering Time Phenotypes

**Figure 1:**
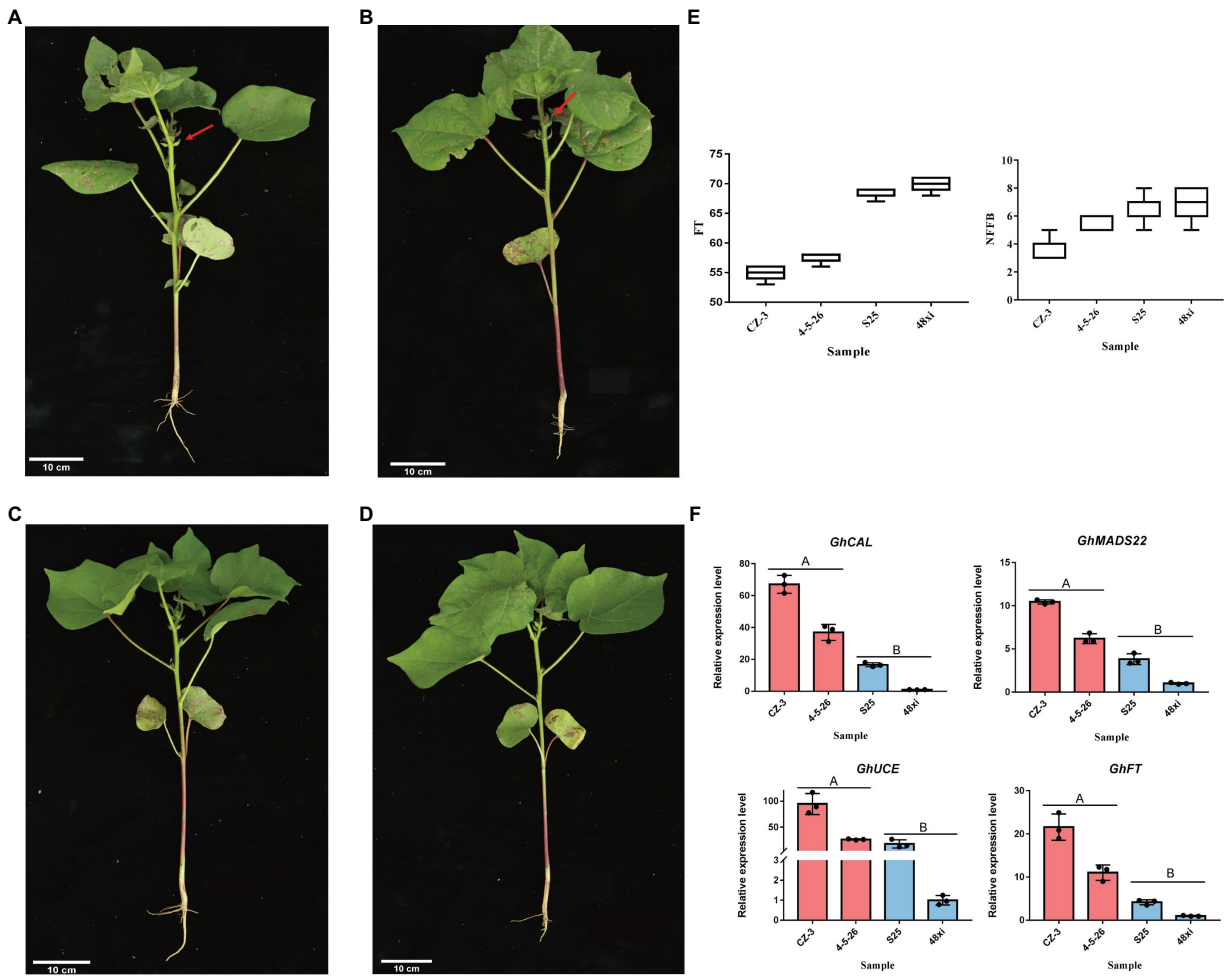
Different phenotypes in the fifth true leaf stage between two early- and two later-flowering cotton varieties. **(A,B)** Early-flowering CZ-3 and 4-5-26. **(C,D)** Later-flowering S25 and 48xi. The arrows point to the flower buds. Scale bars = 10 cm. **(E)** Node of first fruiting branch and flowering times in the four cotton varieties. **(F)** Expression levels of the flowering time regulatory genes GhCAL, GhMADS22, GhUCE, and GhFT in the cotton shoot apex. Values are means ± SD (*n* = 3).

In this study, two elite early-flowering cultivars, CZ-3 and 4-5-26, and two elite later flowering cultivars, S25 and 48xi, were used at the fifth true leaf stage in cotton (Figures 1A–D). Compared with the S25 and 48xi, average node of 3.6–5.3 in first fruiting branch in EFC was lower than LFC. Flowering time was 15 days early in both CZ-3 and 4-5-26 (Figure 1E). Previous study showed that four flowering time genes such as *GhFT*, *GhMADS22*, *GhCAL*, and *GhUCE* promote the cotton flowering (Zhang et al., 2013; Guo et al., 2015; Ma et al., 2018; Cheng et al., 2020). Therefore, we conducted the expression level of those genes in early and late flowering cultivar at fifth leaves stage. Quantitative real-time PCR (qRT-PCR) analysis revealed that the expression level of *GhFT*, *GhMADS22*, *GhCAL*, and *GhUCE* was 6.75-, 10.2-, 67.07-, and 116.77-fold higher in CZ-3 than in 48xi, respectively (Figure 1F). These results suggest that there is a significant transition between early- and later-flowering cotton varieties at the fifth true leaf stage.

### Comparison of miRNAs Involved in Flowering Time Regulation in Cotton

To analyze the fluctuation of endogenous miRNA expression level in early- and late-flowering cotton varieties, we constructed four sRNA libraries from CZ-3, 4-5-26, S25, and 48xi and performed small RNA sequencing. After low-quality reads were removed from the raw data, the most abundant length of miRNA was 24 nt (Supplementary Table S1). This size accounted for over 68%, 69%, 70%, and 70% of the sRNAs in the CZ-3, 4-5-26, S25, and 48xi sequencing libraries, respectively (Supplementary Table S2). Novel miRNAs were identified using the MIREAP software based on their precursors, and the hairpin RNA structures containing sequences by using Mfold software. Minimal folding energy (dG in kcal/mol ≤ −17) was considered as novel miRNAs.

**Figure 2:**
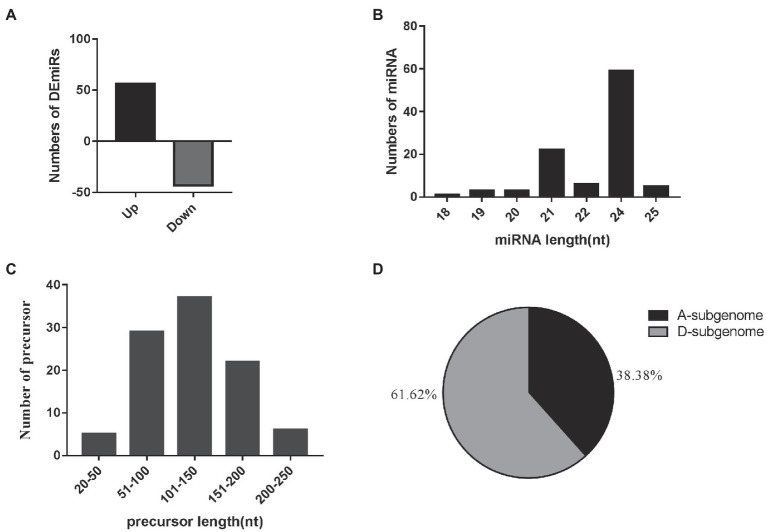
Overview of small RNA sequencing. **(A)** Relative changes in gene expression profiles of the DEmiRs between the early flowering varieties and the later flowering varieties. **(B)** The length distribution of 99 mature miRNAs. **(C)** The length distribution of 99 miRNA precursors. **(D)** Distribution of 99 mature miRNAs in the cotton A- and D-subgenomes.

**Figure 3:**
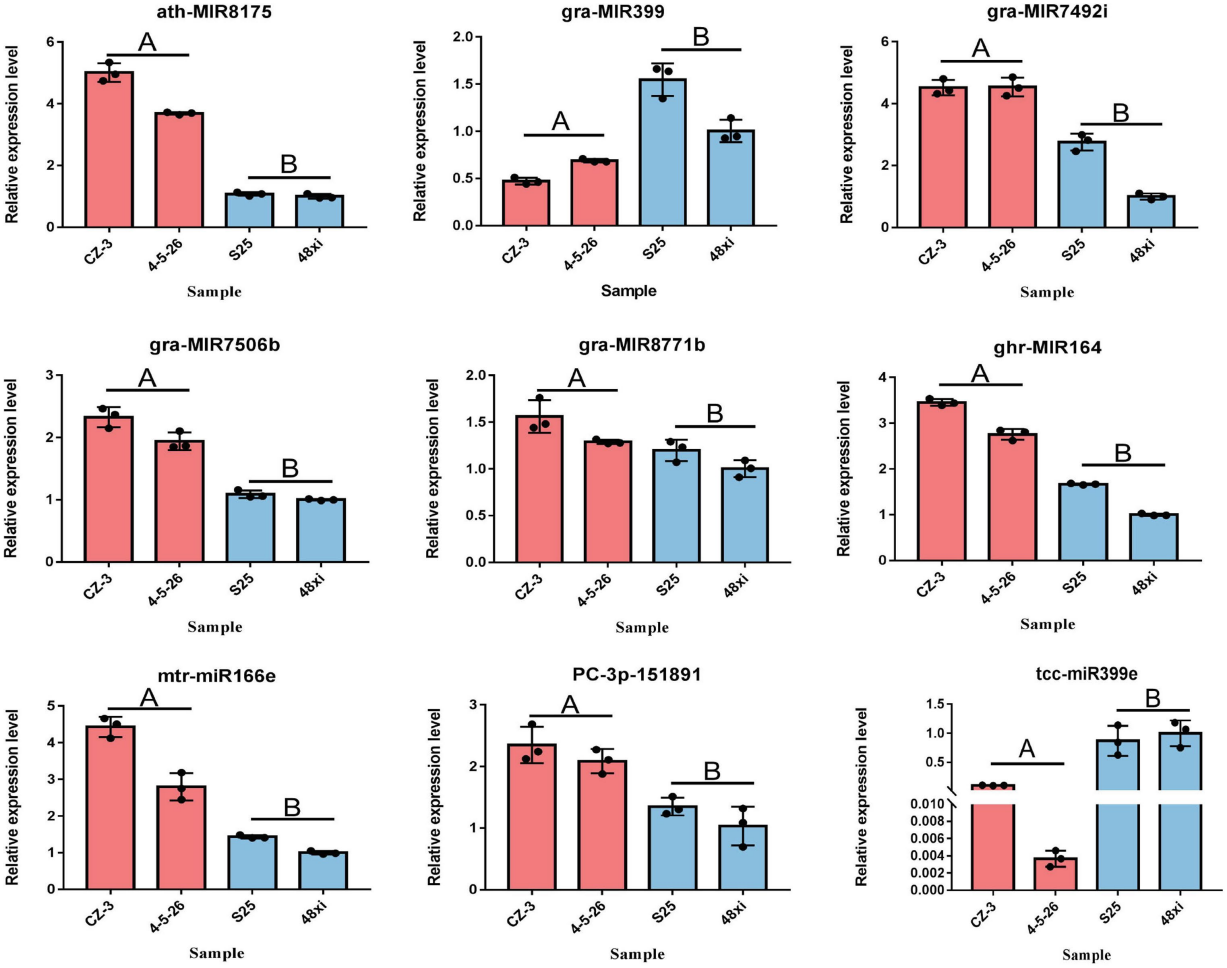
Quantitative real-time PCR verification of seven significantly expressed miRNAs at the fifth true leaf stage in the cotton shoot apex. Values are means ± SD (*n* = 3).

A total of 99 differentially-expressed miRNAs were obtained, including 79 known miRNAs and 20 novel miRNAs (log2 (fold change) >1 or <−1; thresholds of *p* < 0.05). Among these, 56 and 43 miRNAs were significantly up- and downregulated in the two EFCs, respectively (Figure 2A; Supplementary Table S3). Over 59.60% (59) of the mature miRNA sequences were 24 nt in length and 37 precursors were 101–150 length (Figures 2B,C). We also found that 63 miRNAs (including 15 novel miRNAs) map to the D-subgenome (Figure 2D). Besides known miRNA, the lengths of these novel miRNAs varied from 20 to 24 nt. The calculated minimal folding energy ranged from −30.90 to −121 with an average of −70.88 (Supplementary Figure S1). The length of precursor miRNA from 64 to 191 nt (Supplementary Table S3). The correlation assay of differentially expressed miRNA (DEmiRs) between two biological process was strong positive correlation (*R*^2^ > 0.9489) in four varieties (Supplementary Figure S2). To validate these miRNAs, qRT-PCR assays were performed for nine randomly selected miRNAs and consistent the results with miRNA-seq (Figure 3).

Our results showed that seven miRNAs were uniquely expressed in the two EFCs including gra-MIR7506b, two gra-MIR8771b, two gra-MIR8780, and two novel miRNAs. While six miRNAs were only expressed in the two LFCs including tcc-miR398a, two gra-MIR8674b and three novel miRNAs (Supplementary Table S3). Fourteen members of miR6300 family and nine miR7489 family members were significantly upregulated in the two EFCs, whereas nine members of miR399 family were downregulated in the two LFCs. In addition, two conserved miRNAs, miR156 and miR319, which are known to function in flowering time regulation in plants, were significantly downregulated in two EFCs compared with the LFCs, while one miR164 and one miR166 showed the reverse expression pattern (Supplementary Table S3).

### Predicting the Target Genes of Identified miRNAs

**Figure 4:**
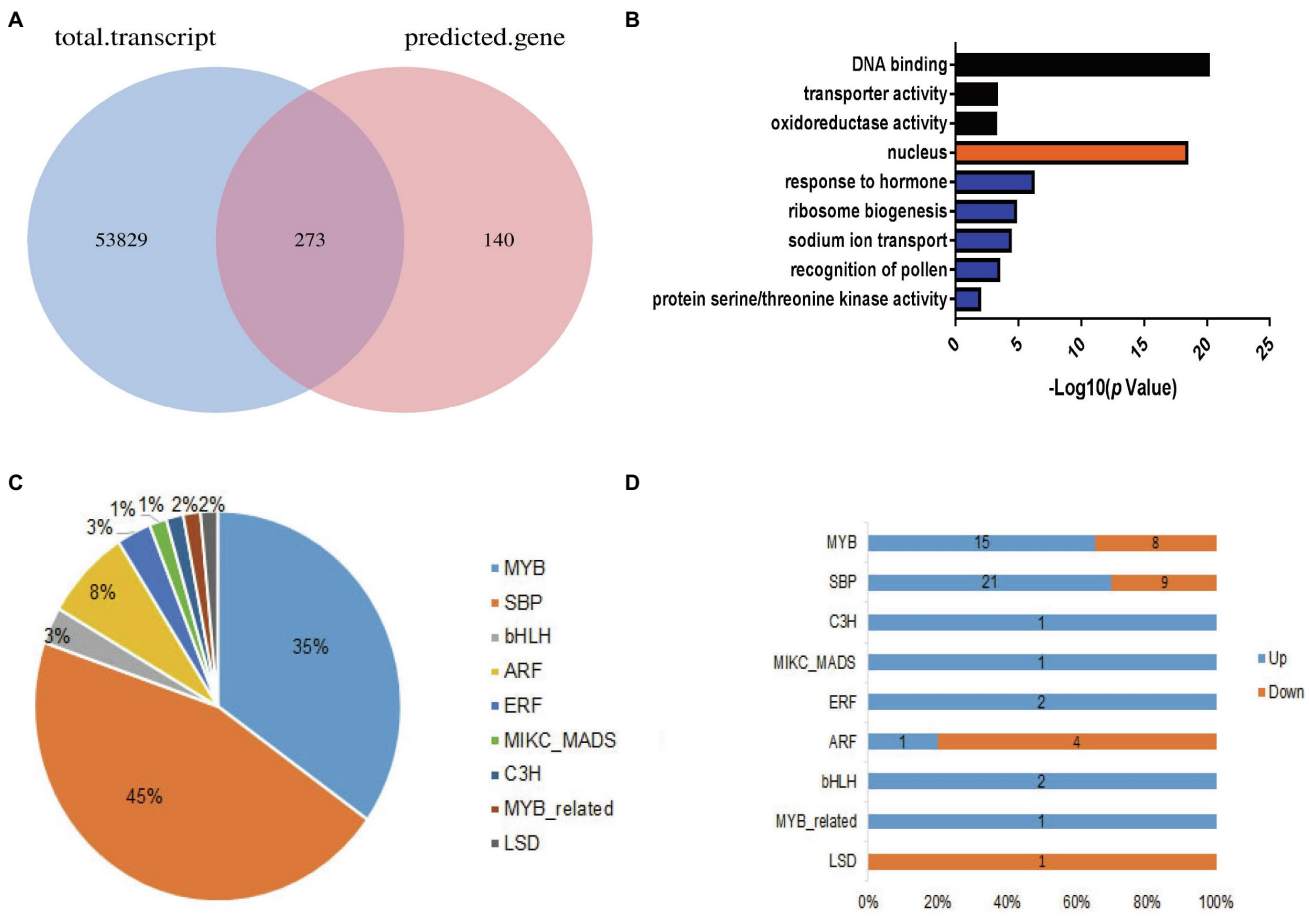
Overview of predicted target miRNA genes. **(A)** Venn diagram showing the overlapping genes between the total transcripts and the predicted target genes. **(B)** Histogram of gene ontology annotation in the three major gene ontology (GO) categories: black, “molecular function”; orange, “cellular component”; and blue, “biological process.” **(C)** Distribution of transcription factor (TF) families in the overlapping genes. **(D)** Distribution of up- and downregulated TF family genes.

To better understand the molecular mechanism of miRNA regulation in cotton flowering time, we performed miRNA target gene prediction using the psRNATarget website.^6^ Combined with our RNA sequencing data, we retained 273 overlapping genes (Figure 4A). To better understand the functions of these overlapping genes, Gene Ontology (GO) analysis was performed for candidate target genes using the CottonFGD database, which provides annotation information for the “biological process,” “molecular function,” and “cellular component” GO categories (see footnote 4). As shown in Figure 4B, the “DNA binding” GO term was over-represented in the “molecular function” category. Consistently, genes in the GO term “nucleuses” were over-represented in the “cellular component” category. “DNA binding function” with 62 genes was predominant in the main “molecular function” category. The GO terms “response to hormone” and “sodium ion transport” represented major terms in the “biological process” category. This further suggests that miRNA target genes functioned in transcription and in response to hormones in cotton.

**Figure 5:**
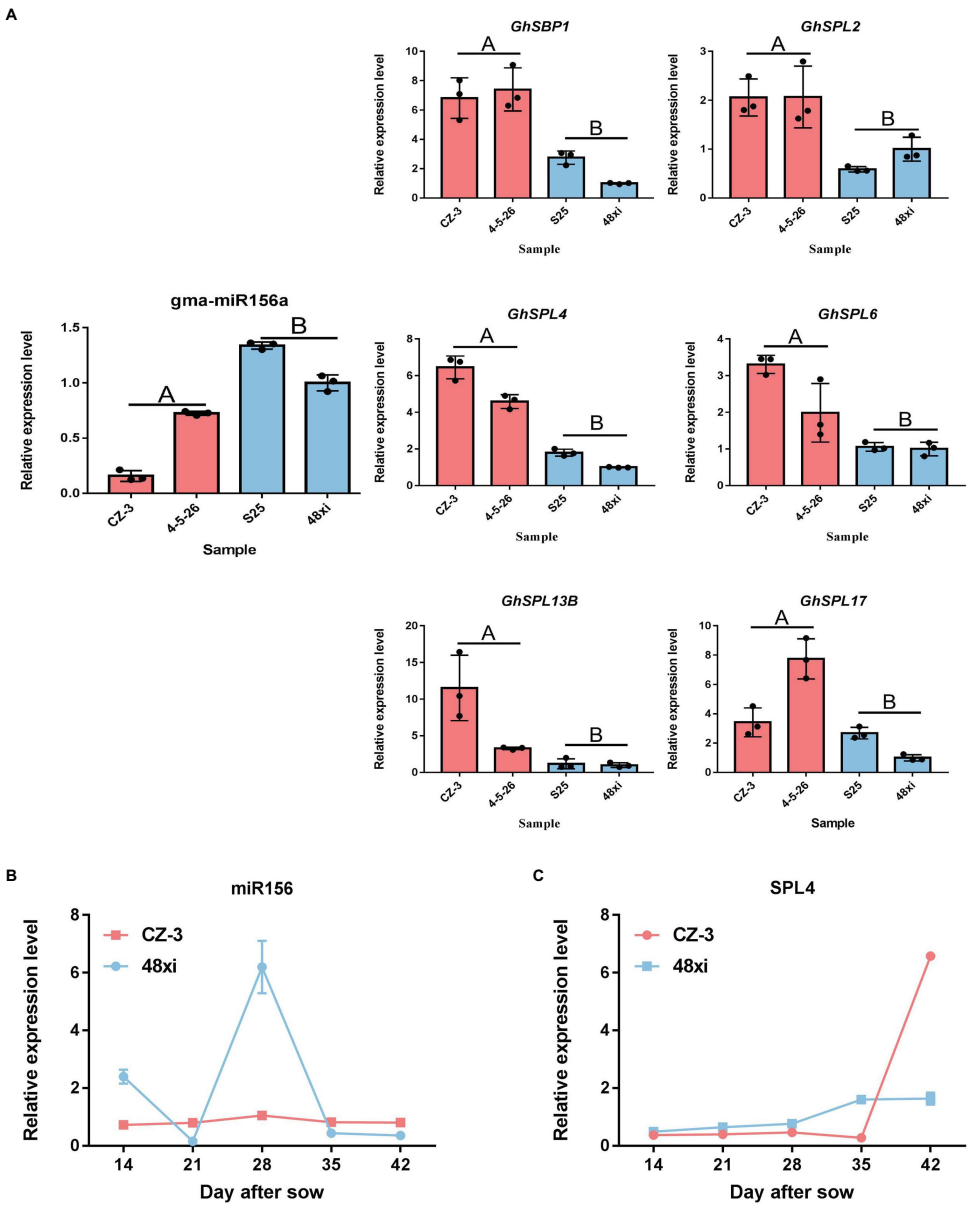
Expression analysis of selected SPL and miR156. **(A)** Quantitative real-time PCR verification of miR156 target gene expression at the fifth true leaf stage in the cotton shoot apex. Values are the means ± SD (*n* = 3). **(B,C)** qRT-PCR analysis of miR156 and SPL4 in CZ-3 and 48xi.

Furthermore, how transcription factors (TFs) influence cotton flowering time, we performed functional analysis of predicted transcription factor genes. The results showed that 66 TF genes were differentially expressed, with 43 upregulated and 23 downregulated in the EFCs. Among (66) TF genes, bHLH (2), ERF (2), C3H (1), and MADS (1) TF genes were significantly enriched in the upregulated DEG group, while LSD (1) TF genes were enriched in the downregulated overlapping group. Except TF families mentioned above, genes encoding SBP-, MYB-, and ARF-type TFs showed both up- and downregulated expression profiles (Figures 4C,D). Interestingly, 30 (45%) of the genes encoding SPL family TFs were significantly upregulated in the EFCs, such as *GH_D01G054600* (*GhSPL4*), *GH_A03G089200* (*GhSPL6*), *GH_D11G041400* (*GhSPL13B*), *GH_A10G019300* (*GhSBP1*), and *GH_A01G147900* (*GhSPL17*); the homologs of these gene in Arabidopsis are targeted by miR156 (Supplementary Table S4). To validate the expression levels in different cotton lines, qRT-PCR assays were performed for the six SPL TF genes which were upregulated in EFC compared with the LFCs, while miR156 expressed the downregulation in EFC (Figure 5A). To further characterize the miR156 and SPL4 expression level during cotton flowering, we analyzed the time-course expression from 14 to 42 days. As shown in Figures 5B,C, the relative expression level of miR156 in 28 days of CZ-3 was 1, while that of 48xi was 6-fold. At the same time, the expression level of SPL4 gene in SAM of CZ-3 was upregulated by 32.5 times from 35 to 42 days after sowing, while the expression of SPL4 in 48xi was only upregulated by 1 time during the same period.

### Genome-Wide Validation of Expression Profiles of Identified miRNA Target Genes

**Figure 6:**
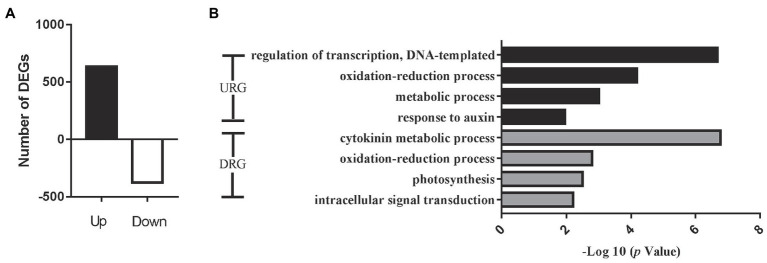
Overview of significantly expressed genes in the fifth true leaf in cotton. **(A)** Changes in gene expression profile between early flowering varieties and later flowering varieties. DEGs, differentially expressed genes. **(B)** Histogram showing GO annotation of the DEGs. URGs, upregulated genes and DRGs, downregulated genes.

To confirm the function of the miRNA target genes involved in cotton flowering regulation, we further conducted RNA sequencing (RNA-seq) analysis to identify the gene expression levels using apical meristems from the EFCs and LFCs. The correlation assay of DEGs between two biological process was strong positive correlation (*R*^2^ > 0.9945) in four varieties (Supplementary Figure S3). A total of 1,008 differentially expressed genes (DEGs; |log2FC| > 1 and value of *p* < 0.05) were identified, where 633 and 375 genes were significantly up- and downregulated in the ELCs, respectively (Figure 6A). Enrichment analysis of all the DEGs identified the significant GO function in “response to auxin” and “cytokinin metabolic process” (Figure 6B). Further analysis showed that the DEGs were also enriched in the GO terms “iron ion binding,” “ADP binding,” and “heme binding.” The 24 genes associated with “iron ion binding” included 15 cytochrome-related genes, 16 genes encoding ADP binding proteins, and 23 genes encoding heme binding proteins. These results exhibited that iron and heme binding activity play a pivotal role in the cotton flowering transition. Except for the above regulation, 46 and 27 genes were associated with oxidoreductase activity and protein dimerization activity, respectively. These results indicated that iron ion, heme, and ADP also function in the control of cotton flowering time.

**Figure 7:**
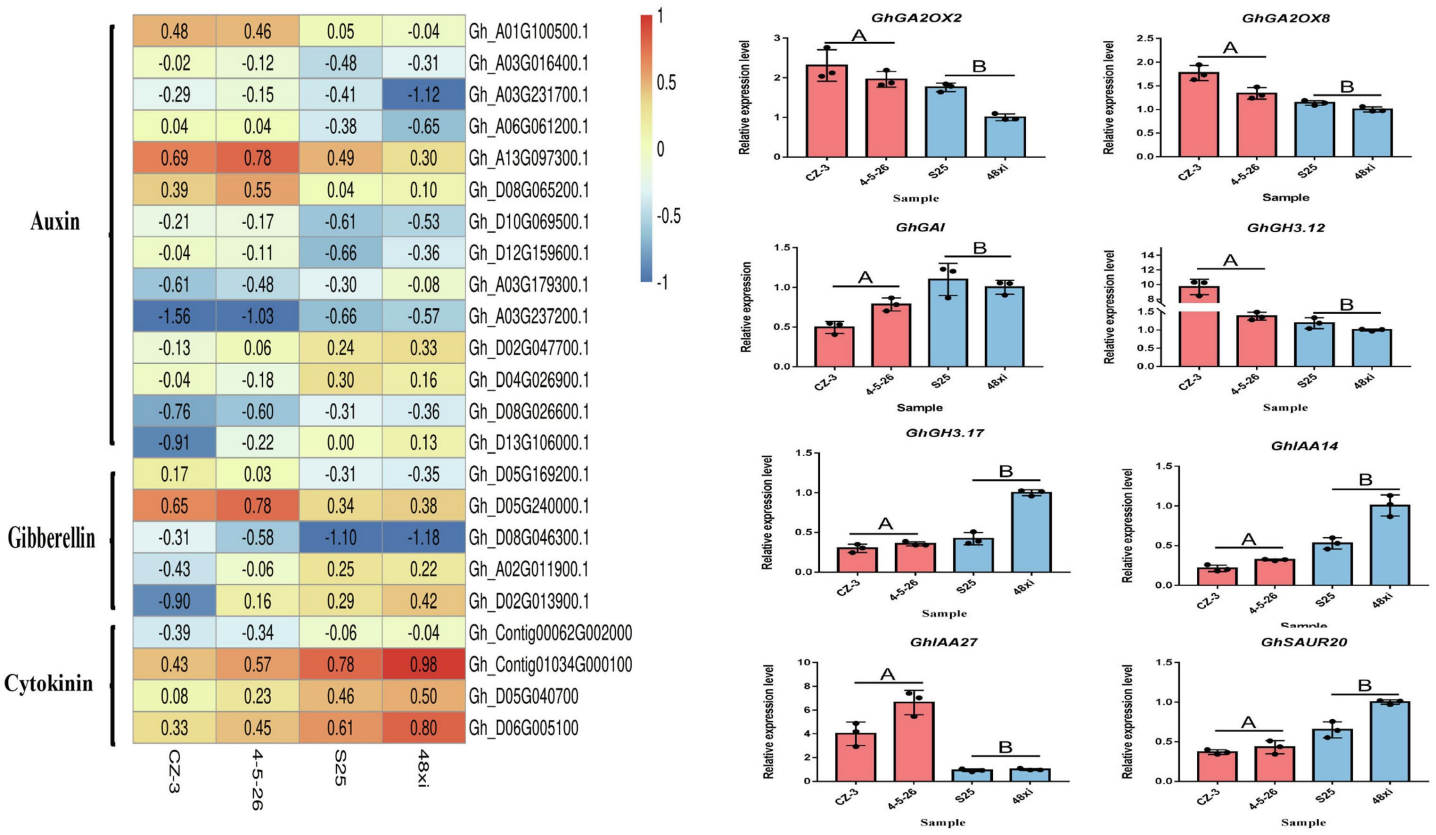
Expression analyses of hormone-related gene in DEG. (A) Expression heat map of hormone-related genes. (B) Quantitative real-time PCR verification of the expression of eight hormone-related genes in the four cotton varieties at the fifth true leaf stage in the shoot apex. Values are means ± SD (*n* = 3).

The phytohormones such as auxin, cytokinin, and gibberellin involve in control of flowering time. The expression of 12 genes was significantly upregulated in the EFCs, and six auxin pathway genes were significantly downregulated in the LFCs. These include seven small auxin upregulated RNA (*SAUR*) members, four auxin-responsive Gretchen Hagen 3 (*GH3*) members, three auxin/indole-3-acetic acid (Aux/IAA) protein family members, and three auxin-binding protein (*ABP*) members. Notably, four cytokinin dehydrogenase (*CKX*) family genes were significantly downregulated in the EFCs. In addition to auxin and cytokinin, gibberellin also functions in cotton flowering time control. Two gibberellin 2-beta-dioxygenase (*GA2OX*) family genes, two *GhGAI* and one gibberellin oxidase showed significant expression in the EFCs (Figure 7A). These results indicated that auxin, cytokinin, and gibberellin function in cotton flowering time control, and the functions of auxin and cytokinin might be diverse. We confirmed the expression level differences between EFCs and LFCs by qRT-PCR and consistent with the result of RNA-seq (Figure 7B). In further study of how flowering marker genes are differentially expressed in the fifth true leaf, 14 genes that participate in cotton flowering time control including *GhGI*, *GhAGL5*, *GhAGL6*, *GhGAI*, *GhAP1*, *GhAGL16*, *GhSOC1*, *GhCOL3*, and *GhCAL*. *GhAGL5*, *GhAGL6*, *GhAP1*, *GhSOC1*, *GhCOL3*, *GhCAL*, and *GhMADS6* showed higher expression levels in the EFCs, which is consistent with previous research. Among these genes, *GhSOC1* and *GhCAL* both showed two-fold increase in expression in the EFCs. Most strikingly, expression of *GhMADS6*, *GhAP1*, *GhAGL5*, and *GhAGL6* increased six-, six-, 10-, and 12-fold in the EFCs, respectively, although *GhGAI* was downregulated by 21-fold compared with the LFCs. These results imply that *GhAGL6*, *GhAP1*, *GhAGL16*, *GhSOC1*, *GhCOL3*, and *GhCAL* may be potential key regulators of flowering time in cotton.

### *GhmiR156* and *GhmiR399e* Negatively Regulated Flowering

**Figure 8:**
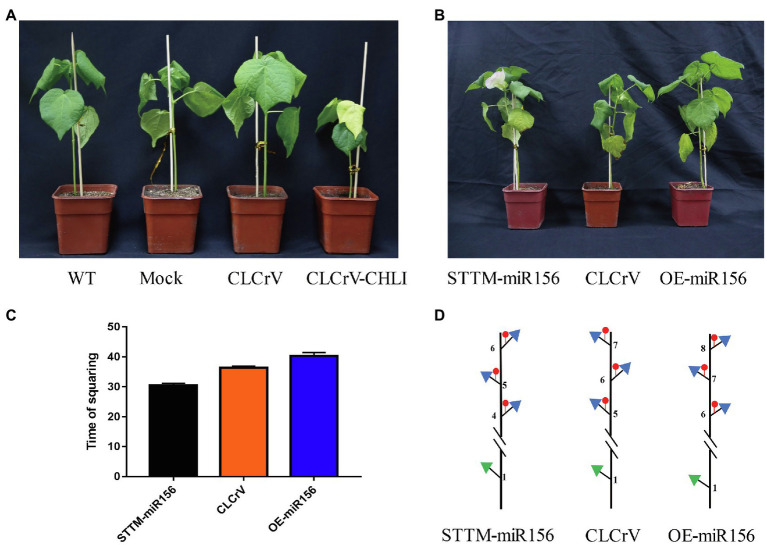
The *GhmiR156* negatively regulated plant flowering. **(A)** Photobleaching phenotype in *CHLI* silenced plant. **(B)** Phenotype of *GhmiR156* overexpressed and silenced cotton. **(C)** Time of squaring described by days after germination (DAG) in overexpressed and silenced plants. **(D)** Sketch map for node position of first fruit branch in overexpressed and silenced plants.

To confirm whether the differentially expressed miRNAs were involved in regulating flowering time, we used the virus-based miRNA silencing (VbMS) strategy to generate Gh-miR156 silenced and overexpressed plants. The OE-miR156 and STTM-miRNA vectors were constructed, containing the fragment of pre-miR156 and a small tandem target mimic (STTM) sequence including two imperfect binding sites separated by a 48 bp spacer, respectively. The positive control, *G. hirsutum* magnesium chelatase subunit I (CHLI) gene was well-silenced, resulting in a photobleaching phenotype in the third true leaf (Figure 8A). Compared with the control plants inoculated with the empty vector (CLCrV) in squaring stage, the silenced plants (STTM-miR156) begun to flower, while the overexpressed ones (OE-miR156) were still in vegetative stage at same time (Figure 8B). The squaring time of CLCrV, STTM-miR156, and OE-miR156 plants was 36, 30.5, and 40.3 DAG (Figure 8C), and the node positions of first fruit branch were 5, 4, and 6 (Figure 8D). These results suggested that Gh-miR156 participated regulating flowering time in cotton.

To determine whether miR399e mediated cotton flowering as well, we performed expression analyses in four cultivars by qRT-PCR. Compared with early flowering cultivars, the expression levels of miR399e of two later flowering cultivars were increased 18.6-fold and the target gene *GhUBC24* reduced expression for 2.36-fold in average. We further introduced miR399e into *Arabidopsis* wild type (WT). The OE-miR399e plants showed the phenotype of delaying flowering compared to WT (Supplementary Figure S4). These results indicated miR399e negatively regulated flowering in cotton.

### Co-expression Network Analysis Uncovers Flowering Gene Interaction Modules

**Figure 9:**
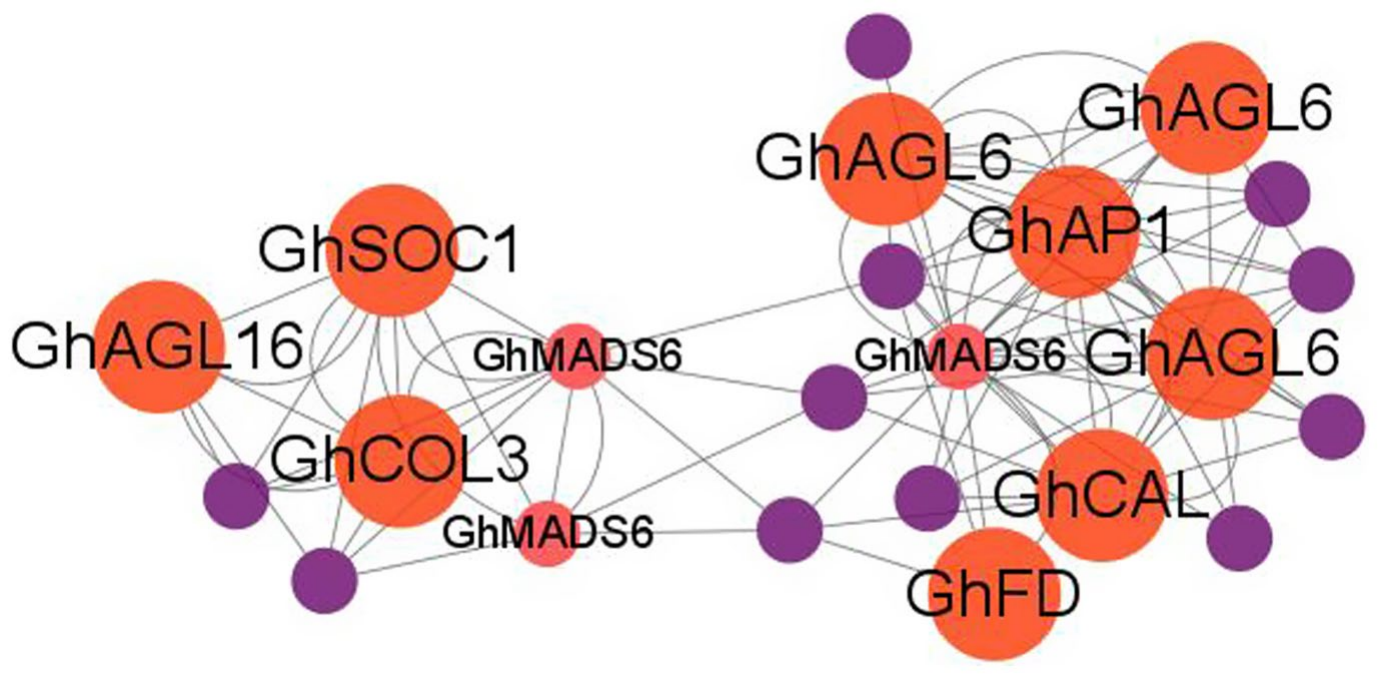
WGCNA analysis of the hub genes in the turquoise module. Red, flowering-related genes and purple, hormone-related genes.

To further describe the miRNA-mediated flowering time regulatory network in cotton, we performed the weighted correlation network analysis (WGCNA) using whole-genome transcriptome data. We identified three modules (blue, turquoise, and brown) that consisted of 920 DEGs which are potentially regulated by SPL TFs (Supplementary Figure S5). The blue module contained 107 nodes and 968 edges, and *GhCAL* and *GhSAUR36* were included in this module. GO analysis revealed that 4 (100%), 4 (80%), and 5 (83.3%) DEGs enriched in “heme binding,” “calcium ion binding,” and “iron ion binding” in the “molecular function” category, respectively, were significantly upregulated in the LFCs compared with the EFCs (Supplementary Figure S6). The brown module contained 61 nodes and 1,449 edges. Three, four, and eight of DEGs were enriched in the “flavin adenine dinucleotide binding,” “oxidoreductase activity,” and “catalytic activity” terms, respectively (Supplementary Figure S6). It is notable that the turquoise module contains 750 nodes and 75,160 edges (Supplementary Table S5). Enrichment analysis of all the DEGs identified 16 “molecular function” and seven “biological process” terms in the turquoise module. Noticeably, 45 of the DEGs were enriched in the “regulation of transcription” biological process term, and 12 DEGs were associated with flowering time pathways (Supplementary Figure S6). These include *GhAP1C*, *GhAGL6*, *GhSOC1*, *GhMADS6*, *GhAP1*, *GhFD*, *GhAGL16*, and *GhCAL* (Figure 9). In addition, four genes associated with auxin signal transduction were identified in the “biological process” category, including *GhIAA14*, *GhIAA27*, *GhIAA29*, and *GhAUX22D*. We also found that “response to auxin process” was enriched in the “biological process” category, and four (66.67%) small auxin upregulated RNA (*SAUR*) genes were upregulated in the EFCs, including *SAUR24*, *SAUR32*, *SAUR50*, and *SAUR72*. These results strongly suggest that auxin and conserved flowering marker genes regulated flowering time in cotton.

## Discussion

MiRNAs, a class of small RNA molecules, have emerged as important regulators of various cellular processes through their interactions with their targets at the transcriptional and post-transcriptional levels (Song et al., 2019). However, the molecular mechanisms involved in the regulation of flowering time in cotton remain unclear. In this study, to better explore the roles of miRNA in flowering time control, we combined sRNA and transcriptome sequencing to identify the relationships between miRNAs and their target genes. In the upland cotton genome, genome-wide bioinformatics analysis predicted 315 miRNAs.^1^ In this study from sequencing analysis of sRNA libraries in four cotton varieties, a total of 186.8 million clean reads included 168 conserved miRNA families and 351 novel miRNAs. We identified 79 conserved miRNAs and 20 novel miRNAs that showed the significant differences in expression (Supplementary Table S3). The sRNA lengths ranged from 18 to 25 nt, and among these, the 24-nt sRNAs were the most abundant in the sequencing libraries, which was similar to previous observations in cotton miRNA sequencing (Li et al., 2012; Hu et al., 2020). It has been reported that many of the 24-nt sRNAs are heterochromatic siRNAs (hetsiRNAs) which mediate transcriptional gene silencing through DNA methylation (RdDM; Lu et al., 2005). Previous studies have shown that flowering-related SNPs are located on the cotton D subgenome; consistent with this notion, 61.62% of miRNAs that showed significant differential expression are located on the D-subgenome (Figure 2D; Ma et al., 2018). However, further studies will be required to verify the relationship between these miRNAs and flowering time.

Among identified miRNAs, more than 10 highly conserved miRNA were identified including miR156, miR159, miR319, miR399, miR7489, miR6300, miR7492, miR8674, miR8771, and miR8780, which is in line with previous study on the development of cotton seeds and fruiting branches (Wang et al., 2015; Sun et al., 2016). Moreover, miR156 was significantly expressed in sRNA sequencing data (Supplementary Table S3). Many studies showed that miR156 expression switches on in the vegetative stage in crops (Chen et al., 2014; An et al., 2015; Aung et al., 2015; Sun et al., 2015; Jia et al., 2017; Zhou et al., 2021). Other studies also stated that miR156 represses the expression of SPL that negatively mediate flowering time (Wang et al., 2009; Wu et al., 2009). It is notable that 9 of 111 miRNAs belong to the miR399 family (Supplementary Table S3), suggesting that there might be additional players in the regulatory pathways. It has been reported that miR399 acts to promote flowering by targeting *PHO2* in response to ambient temperature changes (Kim et al., 2011). It also demonstrated that *PHO2* and *GI* interact to mediate flowering time and phosphate homeostasis in rice (Li et al., 2017). Therefore, it is possible that miR399 may also regulate *PHO2* to influence flowering time in cotton.

Identification of miRNA targets is important to understand miRNA-mediated processes. Bioinformatics analysis revealed 273 target genes including 30 SBP TF genes, of which 21 were upregulated and nine were downregulated in the early flowering varieties (Figures 4A–D). This result suggests that most of the SBP TFs positively regulated flowering progress in cotton. The qRT-PCR results also confirm that *GhPSPL4*, *GhSBP1*, *GhSPL6*, and *GhSPL13B* showed the reverse expression trends compared with miR156 (Figure 5). This result suggests that miR156 potentially repressed *SPL* gene expression in the timing of cotton flowering. A previous study showed that SPL functions as a core regulator of flowering time that positively mediates flowering in Arabidopsis through actions of the SPL-SOC1 module (Jung et al., 2012). Further analysis showed that SPL TFs directly bind to the promoters of *FUL*, *LFY*, and *AP1*, key genes that are active in regulating flowering (Xie et al., 2020). It is notable that except for the SBP-type TFs, MYB-, MIKC_MADS-, and ARF-type TF genes were also enriched in the 273 target genes identified (Figures 4C,D). Previous studies have shown that these types of TFs play an important role in floral organ specificity and the auxin response (Smaczniak et al., 2012; Korasick et al., 2014). This indicated that floral organs and auxin might influence cotton flowering time.

In addition, bioinformatics analysis also identified 920 DEGs with SPL binding sites (GTAC motifs) in their promoters including *GhAP1*, *GhSOC1*, *GhAGL6*, *GhCOL3*, and *GhFD*. WGCNA exhibited that *GhSOC1*, *GhAGL16*, *GhCOL3*, *GhAGL6*, *GhAP1*, *GhCAL*, and *GhFD* were enriched in the turquoise module (Supplementary Table S5). It has been reported that *AGL6*, *SOC1*, *FD*, and *COL3* regulate flowering time in *Arabidopsis thaliana* (Moon et al., 2003; Abe et al., 2005; Li et al., 2008; Yoo et al., 2011; Tripathi et al., 2017; Cheng et al., 2020). The authors also stated the important role of *GhCAL*, *GhAP1*, and *GhSOC1* in mediating cotton flowering (Zhang et al., 2016; Cheng et al., 2020). Hormone actions are intertwined in regulation of various plant growth and developmental processes, and auxin and gibberellin are the most well-studied phytohormones that are necessary for a variety of developmental activities, including flowering (Cheng et al., 2007). Previous studies showed that auxin directly regulates the expression of genes encoding GA metabolic enzymes (O'Neill and Ross, 2002; Frigerio et al., 2006). In addition, auxin affects the stability of DELLA proteins (Fu and Harberd, 2003). Thus, it is expected that the auxin and gibberellin pathways might be important role in regulating flowering time. Consistent with this opinion, *GhSAUR*, *GhIAA*, *GhGH3*, and *GhGAI* genes showed significant differential expression in our study (Figure 7B).

Elucidating the flowering time-associated molecular mechanism becomes the critical biological question that is not only for agricultural significance in shaping juvenile-to-adult transition, but also has significance due to increasing world population and extreme weather events (Xie et al., 2020). Experiments have shown that flowering time in cotton is related to the activities of auxin and gibberellins. Because of its significant influence on plant growth and development, premature flowering can decrease agricultural yield and biomass in major crops and an increase in the length of vegetative phase that could lead to reduce the seed set (Teotia and Tang, 2015). Understanding of molecular mechanism in controlling flowering pathway could have potential applications in many economically important crops.

## Data Availability Statement

The datasets presented in this study can be found in online repositories. The names of the repository/repositories and accession number(s) can be found at: http://www.ncbi.nlm.nih.gov/ PRJNA785082.

## Author Contributions

YZ, YWa, CL, and ZM designed the research. YZ and AM performed the research. YZ, SG, GS, and YWe analyzed the data. CL and RZ wrote the manuscript. All authors contributed to the article and approved the submitted version.

## Funding

This research was funded by the National Natural Science Foundation of China (31771850).

## Conflict of Interest

The authors declare that the research was conducted in the absence of any commercial or financial relationships that could be construed as a potential conflict of interest.

## Publisher’s Note

All claims expressed in this article are solely those of the authors and do not necessarily represent those of their affiliated organizations, or those of the publisher, the editors and the reviewers. Any product that may be evaluated in this article, or claim that may be made by its manufacturer, is not guaranteed or endorsed by the publisher.

## Supplementary Material

The Supplementary Material for this article can be found online at: https://www.frontiersin.org/articles/10.3389/fpls.2022.761244/full#supplementary-material

## Footnotes

^1^ http://www.plantsrnas.org/

^2^ http://omicslab.genetics.ac.cn/psRobot/

^3^ http://www.bioconductor.org/packages/release/bioc/html/DESeq2.html

^4^ https://cottonfgd.org/

^5^ https://horvath.genetics.ucla.edu/html/CoexpressionNetwork/Rpackages/WGCNA/Tutorials/

^6^ http://plantgrn.noble.org/psRNATarget/home
