# Supplementary material for: Insights Into MicroRNA-Mediated Regulation of Flowering Time in Cotton Through Small RNA Sequencing
Source: Front Plant Sci. 2022 Mar 31;13:761244. doi: 10.3389/fpls.2022.761244 (PMC9010036; doi:10.3389/fpls.2022.761244)
Supplement: Supplementary file 1 [file Data_Sheet_1.docx]

**SUPPLEMENTARY DATA**

**Figure S1. The cotton novel miRNA (green) was illustrated and MFE value of stem-loop structure were shown.**


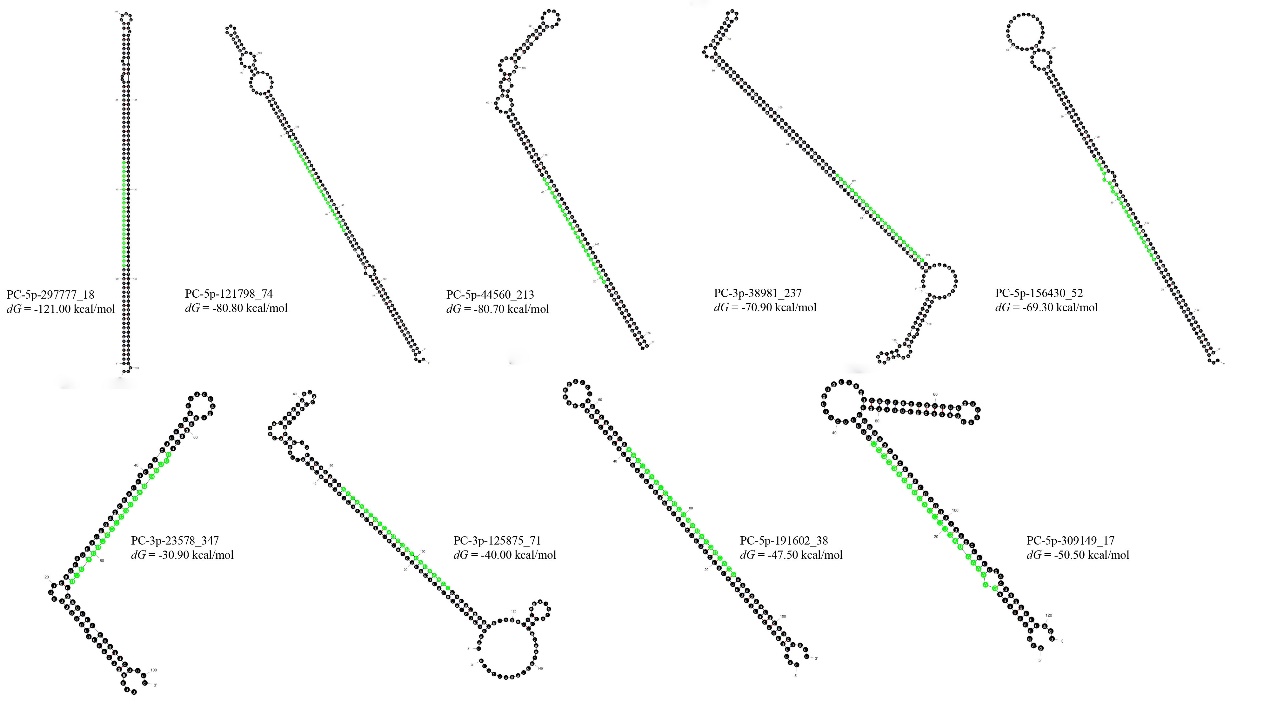


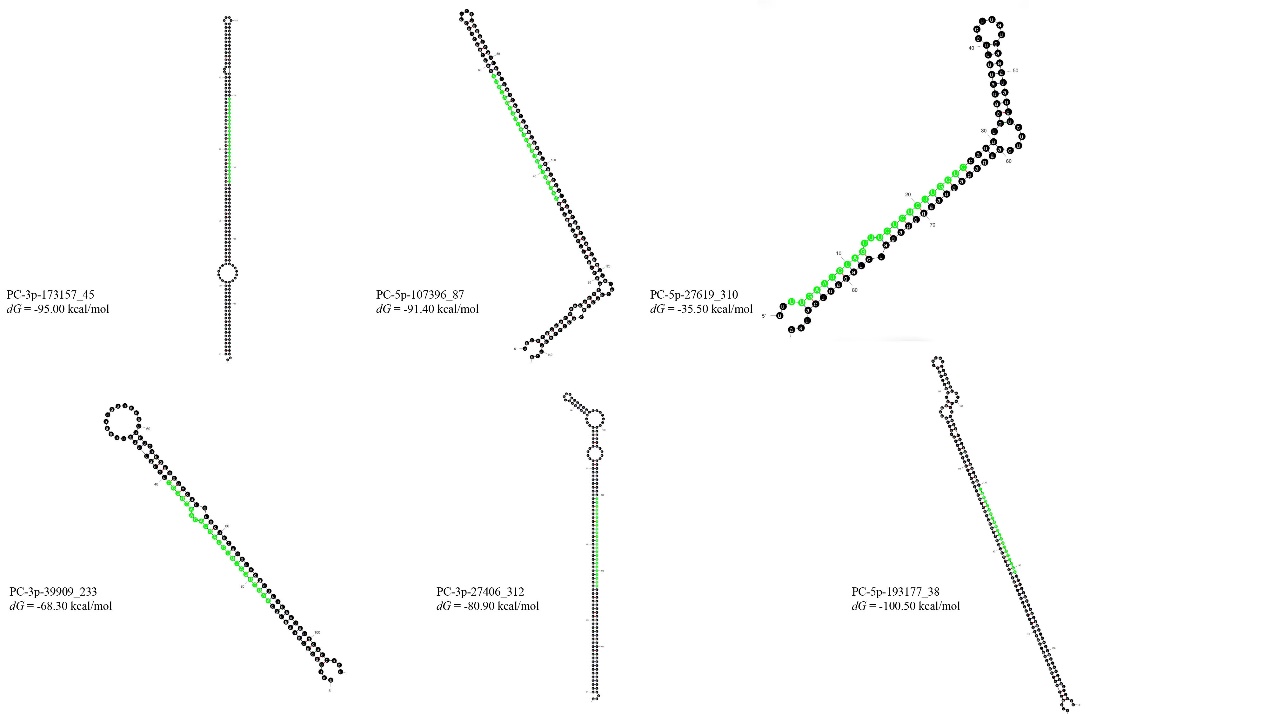


**Figure S2. Correlation assay of DEmiRs between two biological processes in four varieties.**

**
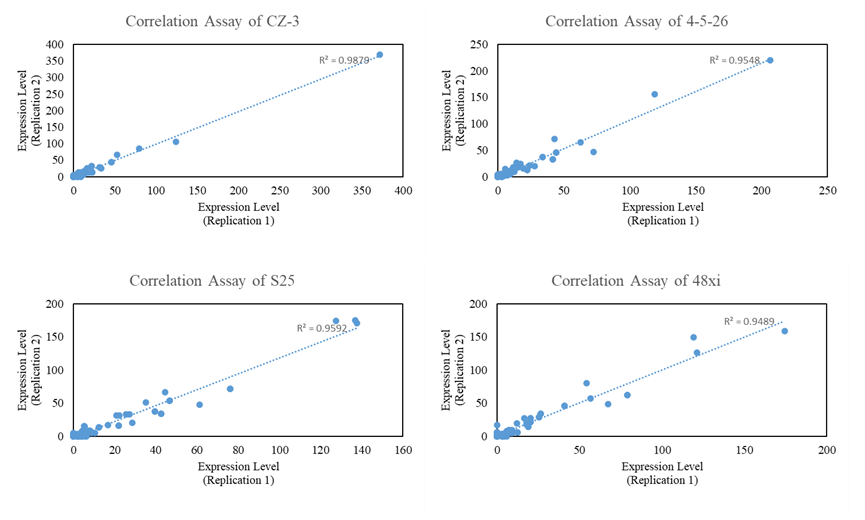
**

**Figure S3. Correlation assay of DEGs between two biological processes in four varieties.**

**
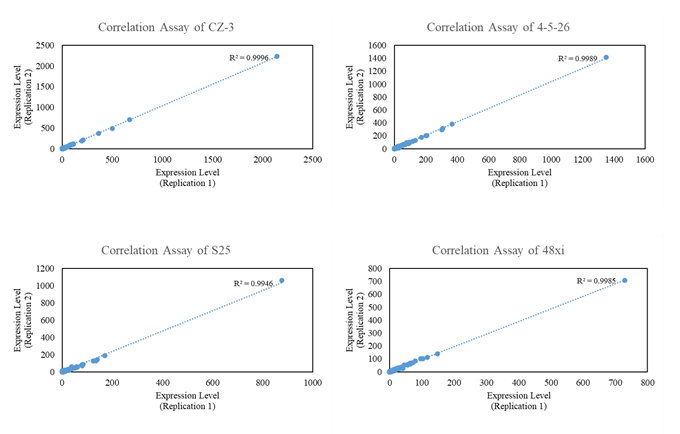
**

**Figure S4. Overexpression (OE) of *GhmiR399e* in *Arabidopsis thaliana* led to early flowering A. The expression level of *GhmiR399e* and target *UBC24*. B. Phenotype of OE of *GhmiR399e* in *Arabidopsis thaliana*. C. Bolting and expression level between trans genetic plant and wild type.**

**
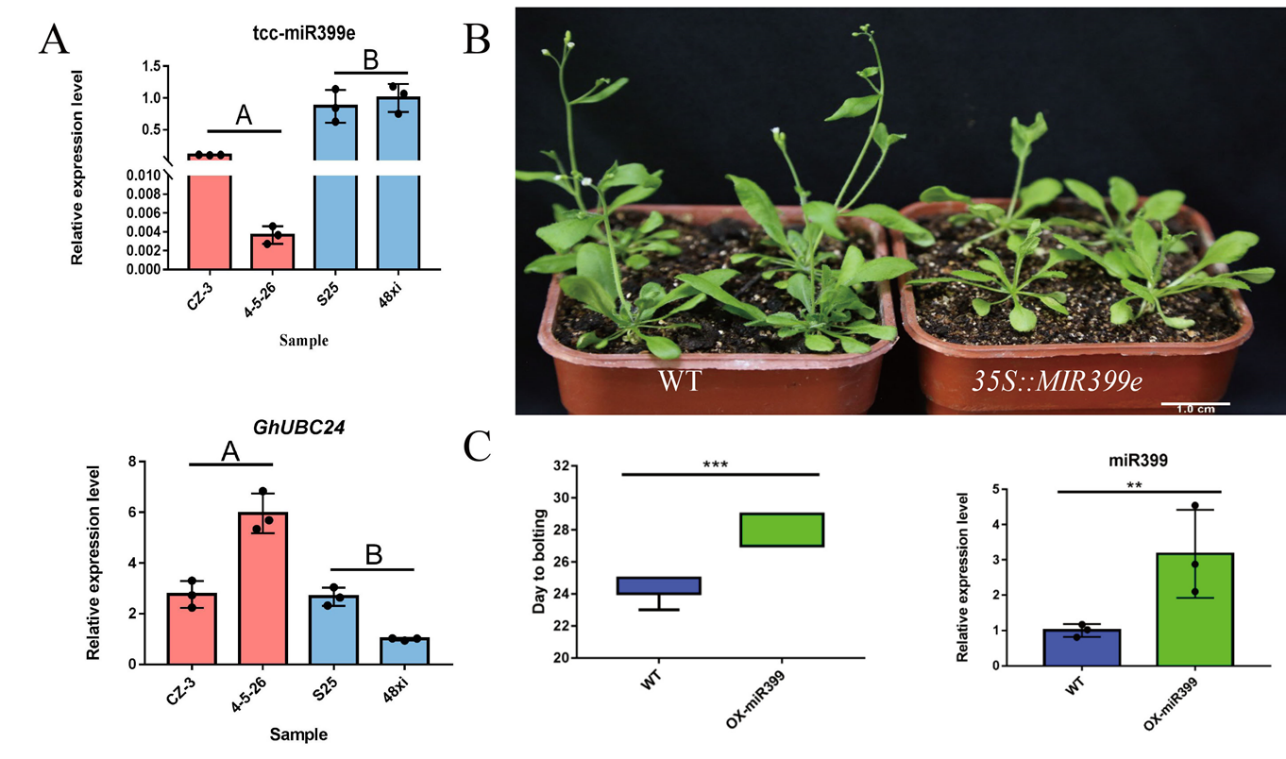
**

**Figure S5. Hierarchical cluster tree showing co-expression modules identified by WGCNA.**


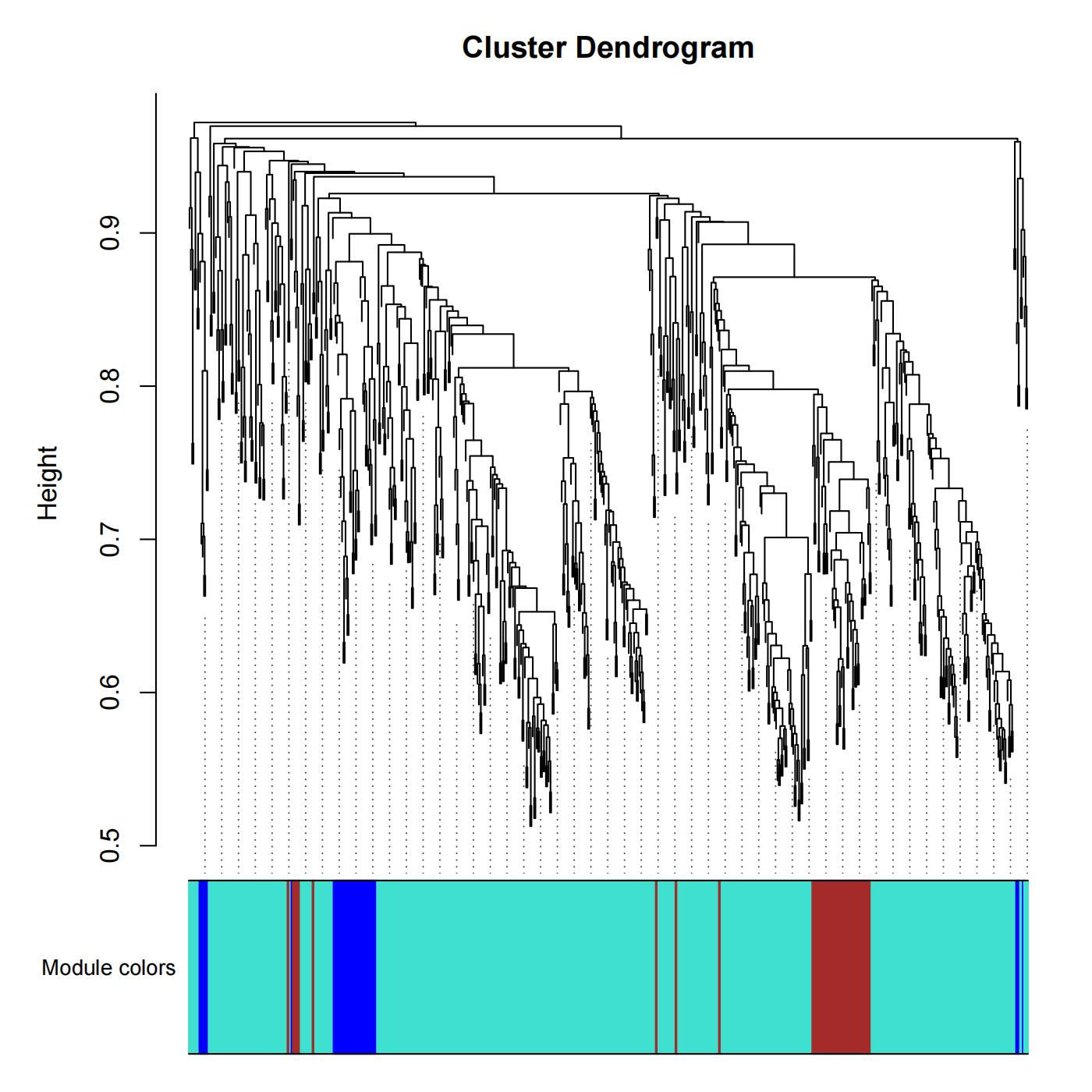


**Figure S6. Histogram of gene ontology annotation in the three major GO categories: Black, ‘molecular function’; Yellow, ‘cellular component’; Blue, ‘biological process’.**

**
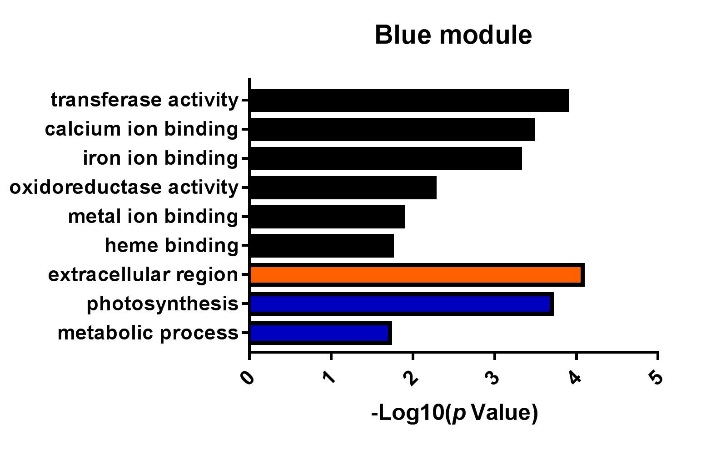
**

**
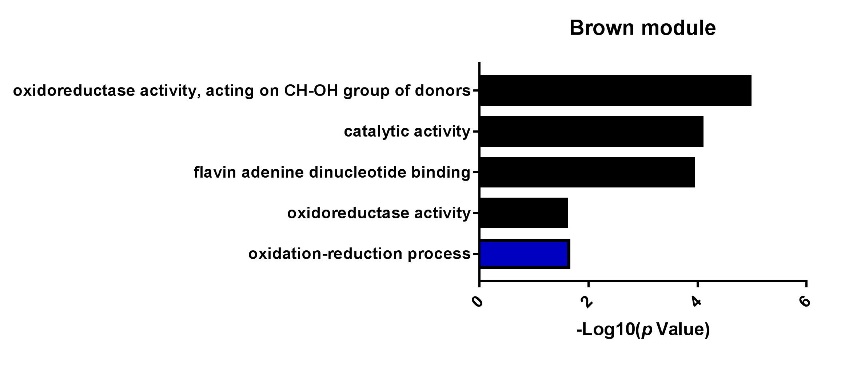
**

**
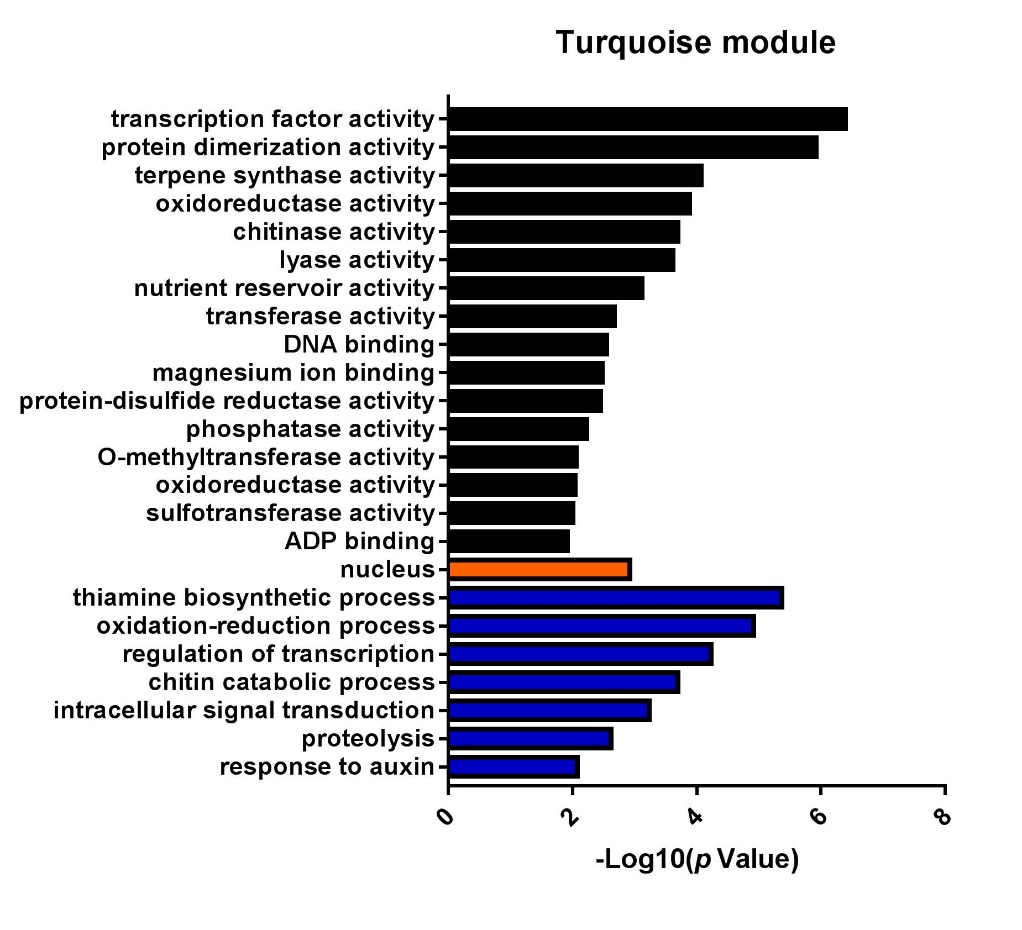
**

**Table S1.** The sRNA reads subjected to several filtering processes.

| Type | Raw reads | 3ADT&length filter | Junk reads | Rfam | mRNA | Repeats | valid reads |
| --- | --- | --- | --- | --- | --- | --- | --- |
| CZ-3 | 12038887 | 1471585 | 98181 | 93650 | 1288467 | 2549 | 9114219 |
| 4-5-26 | 13271027 | 1524305 | 104408 | 130106 | 1429079 | 3098 | 10119337 |
| S25 | 13230839 | 1079150 | 102004 | 106939 | 1575652 | 3479 | 10400751 |
| 48xi | 10485896 | 1078267 | 91098 | 91040 | 1111936 | 2505 | 8139426 |

**Table S2.** Length distribution of total sequenced reads in the four libraries.

| Length (nt) | 18 | 19 | 20 | 21 | 22 | 23 | 24 | 25 |
| --- | --- | --- | --- | --- | --- | --- | --- | --- |
| CZ-3 | 30327 | 60221 | 128191 | 1242743 | 571669 | 464717 | 6262797 | 353554 |
| 4-5-26 | 56816 | 88870 | 157353 | 1289232 | 596782 | 530911 | 7079245 | 320128 |
| S25 | 30624 | 60778 | 132136 | 1378297 | 648688 | 534309 | 7283476 | 332443 |
| 48xi | 41193 | 71909 | 130092 | 1016795 | 489763 | 421568 | 5705994 | 262112 |

**Table S3.** Differentially expressed miRNAs between EFC and LFC.

| miR_name | Length | genomeID | FPKM in CZ-3 | FPKM in 4-5-26 | FPKM in S25 | FPKM in 48xi |
| --- | --- | --- | --- | --- | --- | --- |
| gra-MIR7502e-p5_1ss7TG | 18 | D13 | 9.753769 | 10.02716 | 2.107859 | 0 |
| ath-MIR8175-p5_2ss14TC18AC | 19 | Contig00934 | 16.50991 | 8.883766 | 1.264715 | 0 |
| gma-miR6300_R+1 | 19 | Contig00934 | 27.31616 | 35.72841 | 10.38617 | 9.17282 |
| gma-miR6300_R+1 | 19 | Contig00934 | 27.31616 | 35.72841 | 10.38617 | 9.17282 |
| PC-3p-151891_55 | 20 | Contig00037 | 10.03836 | 5.060244 | 0 | 0 |
| gma-miR6300_R+2_2 | 20 | D03 | 7.82159 | 9.240451 | 1.99007 | 1.472653 |
| gma-miR6300_R+2_1 | 20 | D09 | 7.82159 | 9.240451 | 1.99007 | 1.472653 |
| tcc-miR398a_1ss21GA | 21 | A02 | 0 | 0 | 2.470536 | 4.737913 |
| ppe-miR399a | 21 | A03 | 20.68234 | 44.73217 | 74.05473 | 70.53895 |
| ghr-miR399d | 21 | A05 | 15.07401 | 14.70072 | 55.59296 | 67.1317 |
| seu-miR319_2ss1CT20AG | 21 | A05 | 1.171294 | 4.523554 | 5.959666 | 8.314652 |
| tcc-MIR172e-p5_1ss4TA | 21 | A05 | 0.976078 | 4.523554 | 6.814588 | 6.530519 |
| tcc-miR399b | 21 | A05 | 14.45541 | 17.33751 | 38.25085 | 43.32773 |
| tcc-miR399e | 21 | A05 | 2.481375 | 1.93343 | 29.17775 | 15.45118 |
| tcc-miR399a_1ss21GT | 21 | A08 | 20.68234 | 44.73217 | 74.05473 | 70.53895 |
| ghr-miR399e_1ss7AT | 21 | A10 | 59.87966 | 57.01612 | 150.9476 | 166.6533 |
| ghr-MIR164-p3 | 21 | A13 | 370.2808 | 213.0037 | 156.1453 | 124.0121 |
| gma-miR156a_R+1 | 21 | A13 | 9.737296 | 22.50109 | 50.26442 | 30.28364 |
| mtr-miR166e-5p_L-1R+1_3ss10GT11CT16TC | 21 | D02 | 5.320237 | 5.000242 | 2.107859 | 0 |
| seu-miR319_2ss1CT20AG | 21 | D04 | 1.171294 | 4.523554 | 5.959666 | 8.314652 |
| tcc-MIR172e-p5_1ss4TA | 21 | D05 | 0.976078 | 4.523554 | 6.814588 | 6.530519 |
| tcc-miR399b | 21 | D05 | 14.45541 | 17.33751 | 38.25085 | 43.32773 |
| tcc-miR399g | 21 | D05 | 11.10381 | 20.86102 | 43.2285 | 56.95664 |
| ghr-MIR7488-p3_1ss16AG | 21 | D06 | 2.481375 | 0 | 7.703614 | 6.522045 |
| PC-3p-23578_347 | 21 | D07 | 81.86857 | 63.74977 | 24.44623 | 21.36722 |
| gra-miR8741 | 21 | D11 | 9.469181 | 7.767046 | 3.184113 | 0 |
| gra-MIR399-p3 | 21 | D12 | 30.32676 | 59.76955 | 154.4355 | 134.1871 |
| PC-5p-193177_38 | 21 | D12 | 0 | 0 | 6.135115 | 6.522045 |
| gma-miR156a_R+1 | 21 | D13 | 9.737296 | 22.50109 | 50.26442 | 30.28364 |
| gma-miR828a | 22 | A03 | 18.3033 | 17.35417 | 5.737101 | 6.522045 |
| tcc-miR393a | 22 | A10 | 45.08323 | 37.39848 | 19.16604 | 16.59541 |
| gma-miR6300_R+4 | 22 | Contig00934 | 9.489159 | 10.70052 | 5.561652 | 0 |
| tcc-miR393a | 22 | D04 | 45.08323 | 37.39848 | 19.16604 | 16.59541 |
| gra-MIR8752-p3 | 22 | D11 | 7.16655 | 5.386928 | 2.786099 | 0 |
| fve-miR159c_R+1_1ss1AT | 22 |  | 53.32224 | 39.74527 | 98.35153 | 88.22557 |
| ghr-MIR7489-p5_2ss11GA24CT | 24 | A01 | 3.77849 | 5.700275 | 1.791063 | 0 |
| gra-MIR8674b-p3_2ss11TC20GT | 24 | A02 | 0 | 0 | 2.247972 | 3.854321 |
| gra-MIR8674b-p3_2ss11TC20GT | 24 | A02 | 0 | 0 | 2.247972 | 3.854321 |
| PC-3p-39909_233 | 24 | A03 | 2.961178 | 4.716897 | 6.931143 | 9.198243 |
| PC-5p-85658_114 | 24 | A06 | 6.385687 | 16.48747 | 0 | 0 |
| gma-miR6300_R+6_1 | 24 | A07 | 8.016806 | 7.397023 | 2.645986 | 2.68891 |
| gra-MIR8710b-p3 | 24 | A07 | 0 | 1.160058 | 3.243007 | 3.26526 |
| ghr-MIR7489-p3_2ss20CA24TC | 24 | A08 | 5.356687 | 4.270209 | 1.053929 | 2.061714 |
| gra-MIR7492h-p3_2ss7CT17TG | 24 | A08 | 0 | 1.563407 | 3.688136 | 2.966492 |
| PC-3p-173157_45 | 24 | A08 | 6.1011 | 8.393739 | 1.99007 | 2.987678 |
| ghr-MIR7489-p3_2ss22TC23CT | 24 | A10 | 3.546825 | 2.47012 | 1.053929 | 0 |
| gra-MIR8710b-p3 | 24 | A10 | 0 | 1.160058 | 3.243007 | 3.26526 |
| gra-MIR7492e-p5_2ss17CT24CT | 24 | A11 | 0 | 1.93343 | 6.580244 | 8.297703 |
| PC-3p-27406_312 | 24 | A11 | 4.466475 | 14.56738 | 25.94652 | 18.99402 |
| gma-miR159a-3p_R+3 | 24 | A12 | 2.464903 | 3.823521 | 7.985073 | 6.808101 |
| gra-miR7584e_R+3_1ss19TC | 24 | A12 | 3.742041 | 2.47012 | 0 | 1.472653 |
| bra-miR9560a-5p_1ss13AT | 24 | A13 | 4.645219 | 2.97681 | 12.91437 | 27.00567 |
| ghr-MIR7489-p5_1ss11GA | 24 | D01 | 6.402159 | 7.99039 | 3.254786 | 0 |
| PC-5p-121798_74 | 24 | D01 | 1.952156 | 4.940241 | 6.158673 | 8.314652 |
| gra-MIR7494c-p5 | 24 | D02 | 114.9378 | 137.3267 | 54.67667 | 57.77668 |
| gra-MIR7506b-p3_1ss6GA | 24 | D02 | 9.61498 | 6.786993 | 0 | 0 |
| gra-MIR8674c-p5_2ss2TA19TC | 24 | D02 | 2.411981 | 3.69018 | 1.194042 | 0 |
| PC-3p-38981_237 | 24 | D02 | 12.02346 | 12.3206 | 29.97378 | 20.76968 |
| PC-3p-39909_233 | 24 | D02 | 2.961178 | 4.716897 | 6.931143 | 9.198243 |
| gra-MIR7492i-p3_1ss14CT | 24 | D03 | 29.36715 | 23.90116 | 9.952818 | 1.493839 |
| gra-MIR8771b-p3_2ss16CT23GA | 24 | D03 | 2.802412 | 2.693464 | 0 | 0 |
| gra-MIR8771b-p5_2ss16CT23GA | 24 | D03 | 2.802412 | 2.693464 | 0 | 0 |
| PC-3p-125875_71 | 24 | D04 | 0 | 0 | 5.362644 | 7.11958 |
| ghr-MIR7489-p3_2ss20CA24TC | 24 | D05 | 5.356687 | 4.270209 | 1.053929 | 2.061714 |
| gra-miR7584e_R+3_1ss19TC | 24 | D05 | 3.742041 | 2.47012 | 0 | 1.472653 |
| PC-5p-107396_87 | 24 | D05 | 3.636197 | 4.523554 | 1.264715 | 0 |
| ghr-MIR7489-p3_1ss11GA | 24 | D06 | 6.402159 | 7.99039 | 3.254786 | 0 |
| ghr-MIR7489-p3_2ss4GA17TC | 24 | D06 | 2.713041 | 2.47012 | 0.995035 | 0 |
| ghr-MIR7489-p5_2ss11GA24CT | 24 | D06 | 3.77849 | 5.700275 | 1.791063 | 0 |
| ghr-miR7507_1ss10AG | 24 | D06 | 19.50754 | 20.29433 | 8.266531 | 8.365498 |
| gma-miR6300_R+6_2 | 24 | D06 | 8.016806 | 7.397023 | 2.645986 | 2.68891 |
| gra-miR7484f_L+1R+2_1ss11TC | 24 | D06 | 0 | 15.6541 | 26.74255 | 21.99017 |
| gra-MIR8755-p3_1ss17TA | 24 | D06 | 8.053256 | 6.606988 | 2.529431 | 0 |
| PC-5p-27619_310 | 24 | D06 | 15.57028 | 12.24726 | 38.36741 | 23.45859 |
| PC-5p-27619_310 | 24 | D06 | 15.57028 | 12.24726 | 38.36741 | 23.45859 |
| ghr-miR7509_L-1R+1_1ss2CG | 24 | D07 | 2.802412 | 3.200154 | 0.995035 | 0 |
| gma-miR6300_R+6_1 | 24 | D07 | 8.016806 | 7.397023 | 2.645986 | 2.68891 |
| gra-MIR8780-p3_2ss16AT24AG | 24 | D07 | 4.185394 | 3.363496 | 0 | 0 |
| PC-5p-44560_213 | 24 | D07 | 5.816512 | 5.893618 | 16.78973 | 17.79895 |
| PC-5p-156430_52 | 24 | D08 | 0 | 0 | 5.291971 | 7.708642 |
| PC-5p-191602_38 | 24 | D08 | 0 | 1.93343 | 5.784216 | 6.530519 |
| gra-MIR8773-p3_2ss21CA23GC | 24 | D10 | 3.05055 | 3.466836 | 1.393049 | 0 |
| PC-5p-297777_18 | 24 | D10 | 7.589925 | 7.440362 | 0 | 2.945306 |
| ghr-miR7509_L-1R+1_1ss2CG | 24 | D11 | 2.802412 | 3.200154 | 0.995035 | 0 |
| PC-5p-309149_17 | 24 | D11 | 4.433531 | 0 | 6.907585 | 7.700167 |
| gma-miR159a-3p_R+3 | 24 | D12 | 2.464903 | 3.823521 | 7.985073 | 6.808101 |
| gma-miR6300_R+6_2 | 24 | D12 | 8.016806 | 7.397023 | 2.645986 | 2.68891 |
| gra-MIR8674c-p5_2ss2TA19TC | 24 | D12 | 2.411981 | 3.69018 | 1.194042 | 0 |
| gra-MIR8773-p3_2ss21CA23GC | 24 | D12 | 3.05055 | 3.466836 | 1.393049 | 0 |
| gra-MIR8780-p3_2ss16AT24AG | 24 | D12 | 4.185394 | 3.363496 | 0 | 0 |
| PC-5p-107396_87 | 24 | D12 | 3.636197 | 4.523554 | 1.264715 | 0 |
| bra-miR9560a-5p_1ss13AT | 24 | D13 | 4.645219 | 2.97681 | 12.91437 | 27.00567 |
| ghr-MIR7489-p3_2ss4GA17TC | 24 | D13 | 2.713041 | 2.47012 | 0.995035 | 0 |
| gra-MIR7492h-p3_2ss7CT17TG | 24 | D13 | 0 | 1.563407 | 3.688136 | 2.966492 |
| gma-MIR6300-p5_2 | 25 | A01 | 18.05166 | 13.63066 | 1.264715 | 5.040918 |
| gma-MIR6300-p3 | 25 | A05 | 18.05166 | 13.63066 | 1.264715 | 5.040918 |
| gma-miR6300_R+7 | 25 | A09 | 8.370787 | 5.313589 | 2.669543 | 1.493839 |
| gma-miR6300_R+7 | 25 | A12 | 8.370787 | 5.313589 | 2.669543 | 1.493839 |
| gma-MIR6300-p5_1 | 25 | D05 | 18.05166 | 13.63066 | 1.264715 | 5.040918 |

Table S6. Primers used for qRT-PCR experiments.

| Primer name | Sequence (5ʹ‒3ʹ) |
| --- | --- |

| gra-MIR7506b-p3-reverse p | GTCGTATCCAGTGCAGGGTCCGAGGTATTCGCACTGGATACGACATCGTT |
| --- | --- |
| gra-MIR7506b-p3-qPCR-F | CGCGAAACTCCATTACCTCTCT |
| gra-MIR8771b-p5-reverse p | GTCGTATCCAGTGCAGGGTCCGAGGTATTCGCACTGGATACGACATCGTT |
| gra-MIR8771b-p5-qPCR-F | CGCGAAACTCCATTACCTCTCT |
| PC-3p-151891_55-reverse p | GTCGTATCCAGTGCAGGGTCCGAGGTATTCGCACTGGATACGACTTAGAT |
| PC-3p-151891_55-qPCR-F | CGCGCGCTTTCTACCATACT |
| ath-MIR8175-p5-reverse p | GTCGTATCCAGTGCAGGGTCCGAGGTATTCGCACTGGATACGACAGGCGG |
| ath-MIR8175-p5-qPCR-F | CGCGCGGTGGTAAGTATTC |
| gra-MIR399-p3-reverse p | GTCGTATCCAGTGCAGGGTCCGAGGTATTCGCACTGGATACGACCAGGGC |
| gra-MIR399-p3-qPCR-F | CGCGTGCCAAAGGAGAGTT |
| reverse-miR166e-P | GTCGTATCCAGTGCAGGGTCCGAGGTATTCGCACTGGATACGACGCCTCG |
| mtr-miR166e-F | GCGGAATGTTGTTTGGCC |
| reverse-miR164-P | GTCGTATCCAGTGCAGGGTCCGAGGTATTCGCACTGGATACGACGGTGGA |
| ghr-MIR164-F | CGCACGTGCTCCCCTTC |
| reverse-miR156-P | GTCGTATCCAGTGCAGGGTCCGAGGTATTCGCACTGGATACGACAGTGCT |
| ghr-MIR156-F | GCGCGTGACAGAAGAGAGTG |
| reverse-miR399e-P | GTCGTATCCAGTGCAGGGTCCGAGGTATTCGCACTGGATACGACCGGGGC |
| ghr-MIR399-F | CGCGTGCCAATGGAGATTT |
| Universe-qPCR-R | AGTGCAGGGTCCGAGGTATT |
| UBQ7-F | GAAGGCATTCCACCTGACCAAC |
| UBQ7-R | CTTGACCTTCTTCTTCTTGTGCTTG |
| FT-qPCR-F | CCAACGGTCGGTATCCATCG |
| FT-qPCR-R | GATCCACTCTCCCTCTGGCA |
| GhCAL-qPCR-F | CCAATATCCATTTTTGTTTCAGT |
| GhCAL-qPCR-R | TACCTCTACCCATATTTTGTTTT |
| SPL4-qPCR-F | GGTGATCATGGTTTCCCAGA |
| SPL4-qPCR-R | ACAGAAGCGTTGCCTGAGAC |
| GhMADS22-qPCR-F | GGATCCTCTCCATGCAAGAATCT |
| GhMADS22-qPCR-R | GCATAAGAATATCTTTCATACCGTT |
| GhUBC-qPCR-F | AGCGGATCTTGAAGGAGCTCA |
| GhUBC-qPCR-R | CTAGAAACACTCCACCGGCAT |
| GA2OX2-qPCR-F | CGCTAGGCATAGAGCAATGG |
| GA2OX2-qPCR-R | CCCCAAGCGTAAGGAGTAGG |
| GA2OX8-qPCR-F | TAGTGAAGGATGGGCAATGG |
| GA2OX8-qPCR-R | TCTCATACGATGGGCAAAGG |
| GH3.12-qPCR-F | CCTGTCAACGCCGAAACTCT |
| GH3.12-qPCR-R | GCTTGTTGTGGCAGCTCTTG |
| IAA27-qPCR-F | TCGGGGAGTGCAGTGGGAAA |
| IAA27-qPCR-R | CAGCATGGCCCTTTGAAGCTAA |
| GAI-qPCR-F | GGGGGAGGAAGAAGAGTGTTTGATTTTAG |
| GAI-qPCR-R | AGGCAGCTACTCGACTCAATGT |
| IAA14-qPCR-F | TGGGGAGTTATGGATGTGAAGGAA |
| IAA14-qPCR-R | TGAGTCAACAAACATCTCCCATGG |
| SAUR20-qPCR-F | CTAAACTGTTTGCAAATCCAGCAGC |
| SAUR20-qPCR-R  Pre-miR156-F  Pre-miR156-R  STTM-156-infusion-F  STTM-156-infusion-R  VIGS-premiR156-infusion-F  VIGS-premiR156-infusion-R  Pre-miR399-F  Pre-miR399-R  35s-399infusion F  35s-399infusion R | CTAGTGCAAGCTTGATGTGAGATCAAC  gttgTTGACAGAAGATAGAGAGC  gatGATGACAGAAGCATAGAGAGC  ATGCCTGCAGACTAGTGTGCTCTCTATCTACTTCTGTCA  TAGGGGCGCGCCTTAATTAATTGACAGAAGTAGATAGAGAGCA  ATGCCTGCAGACTAGTGTTGTTGACAGAAGATAGAGAGC  TAGGGGCGCGCCTTAATTAAGGTGATGACAGAAGCATAAAGAGC  TGAGGAATTACAGGGCAACTC  TGAGTGAATGGCAGGGCAAATCTCCTTTGGC  ATGCCTGCAGACTAGTTGAGGAATTACAGGGCAACTCTCC  TAGGGGCGCGCCTTAATTAATGAGTGAATGGCAGGGCAAATC |
